# Structural Relationships Among Physical Activity, Sleep, and Resilience: A Multi-Group Structural Equation Modeling (SEM) Study Among Physically Weak College Students

**DOI:** 10.3390/healthcare14152255

**Published:** 2026-07-23

**Authors:** Hesong You, Chengfei Bao, Yunchen Meng, Sanjun Yang, Ruijian Fang, Wang Yang, Wenti Jiao

**Affiliations:** Department of Physical Education and Research, China University of Mining and Technology-Beijing, Beijing 100083, China

**Keywords:** physical activity, sleep quality, resilience, multi-group structural equation modeling, physically weak students

## Abstract

**Background:** Resilience is a critical psychological resource for university students to cope with stress and maintain mental health; however, students with physical weaknesses may be at greater risk due to physiological limitations. This study aimed to use multi-group structural equation modeling (SEM) to examine the associations among physical activity (PA), sleep quality, and resilience. **Methods:** A cross-sectional study was conducted using stratified cluster sampling among 2040 students from four universities in Beijing. The participants had a mean age of 19.03 ± 0.94 years and included 1144 men (56.1%) and 896 women (43.9%). Participants were categorized into the General Sample (*n* = 1504, 73.7%), Fitness-Remediation Sample (*n* = 341, 16.7%), and Rehabilitation Sample (*n* = 195, 9.6%). PA, sleep quality, and resilience were assessed using the International Physical Activity Questionnaire—Short Form (IPAQ-SF), Pittsburgh Sleep Quality Index (PSQI), and Connor–Davidson Resilience Scale (CD-RISC), respectively. Multi-group SEM was used to estimate direct and indirect associations and to compare structural paths across groups. **Results:** In the total sample, PA was positively associated with resilience and negatively associated with poorer sleep quality. Sleep quality showed a significant indirect association between PA and resilience, accounting for 18.2% of the total effect. Multi-group analyses suggested between-group differences in the PA→PSQI path: it was significant only in the General Sample but not in the Fitness-Remediation or Rehabilitation Sample. The direct PA → resilience path remained statistically significant across all three groups. **Conclusions:** University mental health programs should avoid a one-size-fits-all approach and instead adopt stratified, tailored interventions. For physically weak students, adaptive PA combined with targeted sleep-support strategies may be considered as part of resilience-related health promotion.

## 1. Introduction

Resilience is defined as a critical trait enabling individuals to achieve positive adaptation and functional recovery through psychological and behavioral regulation when confronting stress, adversity, or significant setbacks [1]. In the context of college students’ psychosomatic development, resilience plays a pivotal role, serving as a key protective factor in buffering against mental disorders and maintaining emotional equilibrium [2]. The UNESCO Education 2030 Framework for Action highlights that cultivating resilience and adaptability in learners constitutes a core competency of higher education in the 21st century [3], thereby highlighting its importance for students’ long-term development.

However, for physically weak students, the presence of chronic diseases, poor physical fitness, or obesity imposes a significantly higher risk of physiological functional limitations [4]. These students are characterized not only by lower levels of daily physical activity (PA) but also by a psychological vulnerability that is increasingly drawing attention in academia. Data from the American Psychological Association (APA) indicate that over 60% of college students have been diagnosed with at least one mental health issue [5], highlighting the severity of the situation. Meng et al. (2025) based on a survey of 1248 students, revealed that relative to their healthy peers, physically weak individuals exhibited higher detection rates of depression, anxiety, and psychological stress, alongside more limited reserves of resources for psychological recovery [6]. Consequently, exploring modifiable factors associated with resilience in physically weak students has practical implications.

In recent years, PA has been widely recognized as a modifiable health behavior that is positively associated with individual resilience [7,8]. The goal-oriented nature and challenging tasks inherent in sports provide continuous opportunities for practice, enabling individuals to gradually form stable, positive coping patterns through repeated experiences of success [9]. Specifically, participation in collective or competitive sports may be associated with greater self-efficacy and competence; this “I can do it” belief may also be linked to greater persistence and resilience in broader life contexts [10]. Simultaneously, PA may also be associated with psychological resources such as social connectedness and peer support [11,12]. In summary, PA may represent a practical and modifiable context associated with resilience development and may serve as a useful entry point for mental health promotion in higher education.

Moreover, PA has been associated with better sleep quality, which may serve as a crucial bridge connecting PA and emotional outcomes [13]. Previous studies have suggested that regular PA may be related to sleep through circadian and hormonal pathways, including circadian rhythm regulation and melatonin secretion [14]. High-quality sleep may be an important correlate of resilience-related psychological functioning [15]. A meta-analysis by Scott et al. further suggested that sleep quality may partly account for the association between individual behavior and mental health [16]. For physically vulnerable college students, however, sleep quality problems may be more pronounced and may be negatively associated with resilience. Therefore, thoroughly examining the indirect association involving sleep quality between PA and resilience among physically vulnerable students, and clarifying the intrinsic associations among these three variables, is of great significance for formulating targeted mental health promotion schemes.

Based on the aforementioned background, the present study employs a multi-group structural equation modeling (SEM) approach to examine group differences in the association between PA and resilience among physically weak and general college students, with a specific focus on the distinct performance of sleep quality within the mediating pathway. The findings are expected to provide a theoretical basis for developing targeted health-promotion strategies for physical and mental health and offer practical guidance for physical and mental health education in higher education institutions.

## 2. Materials and Methods

### 2.1. Participants

A stratified cluster sampling method was used to recruit freshmen and sophomore students from four universities in Beijing. All participants were first- or second-year undergraduate students aged 18–20 years. The stratification was based on the type of physical education (PE) course in which students were enrolled, as these course types reflected students’ physical status and PE participation arrangements within the university PE system. Because students with physical weakness were concentrated in relatively small and specific PE course groups, all available rehabilitation PE classes and fitness-remediation PE classes in the four universities were invited to participate to ensure adequate representation of the Rehabilitation Sample and Fitness-Remediation Sample. In contrast, students in general PE classes were more numerous; therefore, as many eligible students from general PE classes as possible were recruited, based on the actual course arrangements and feasibility of data collection at each university, to provide the General Sample as a reference group.

The sampling procedure was as follows: First, all students enrolled in rehabilitation PE classes across the four universities were invited to participate to form the Rehabilitation Sample. These classes included students with chronic illnesses, injuries, inability to participate in regular exercise, or restricted mobility, comprising approximately 240 students. Second, all available fitness-remediation PE classes were invited to participate to form the Fitness-Remediation Sample. These classes included students who had failed the university physical fitness test, comprising approximately 400 students. Third, eligible students from general PE classes were recruited as extensively as possible across the four universities to form the General Sample, comprising approximately 1800 students. This sampling strategy resulted in unequal group sizes, reflecting the actual distribution of PE course types and physically weak students in the university PE system, as well as the feasibility of recruiting students from different PE course categories, rather than random allocation.

A total of 2260 questionnaires were distributed and returned. Invalid questionnaires were excluded based on the following criteria: >10% missing values, failure on reverse-item checks, and clearly patterned responding. Finally, 2040 valid questionnaires were retained. The final sample consisted of 1504 students in the General Sample, 341 in the Fitness-Remediation Sample, and 195 in the Rehabilitation Sample. The sample included 1144 men and 896 women; 1015 freshmen and 1025 sophomores. Participation was voluntary, and written informed consent was obtained from all participants prior to data collection.

### 2.2. Measurement Tools

#### 2.2.1. Basic Information

The questionnaire collected basic demographic and anthropometric information, including sex, age, ethnicity, height, weight, body mass index (BMI), and PE course participation. For students in the Rehabilitation Sample, detailed health-status information was also collected.

#### 2.2.2. Physical Activity

The International Physical Activity Questionnaire Short Form (IPAQ-SF) was used to assess PA among university students and is widely used in public health and epidemiological research [17,18]. The questionnaire contains seven items covering vigorous-intensity activity, moderate-intensity activity, walking, and sitting time, and captures the number of days and duration for each activity category over the past seven days. After data collection, responses were cleaned and processed strictly following the IPAQ guidelines: (1) records were excluded if the sum of moderate-to-vigorous PA, walking, and sitting time exceeded 960 min per day, and (2) any reported duration >180 min per day for vigorous activity, moderate activity, or walking was truncated to 180 min per day [19]. In the present dataset, approximately 0.5% of participants required truncation of extreme PA values. Given this very small proportion, the influence of IPAQ-SF truncation on the overall PA distribution was expected to be limited. Weekly energy expenditure was then calculated according to the IPAQ-SF scoring protocol, and PA level was categorized into low, moderate, and high [20].

#### 2.2.3. Sleep Quality

Sleep quality was assessed using the Pittsburgh Sleep Quality Index (PSQI), one of the most widely used sleep measures internationally with established reliability and validity across diverse populations and cultural contexts [21,22]. The PSQI evaluates sleep across seven interrelated components: (1) subjective sleep quality, (2) sleep latency, (3) sleep duration, (4) habitual sleep efficiency, (5) sleep disturbances, (6) use of sleep medication, and (7) daytime dysfunction. Each component is scored from 0 to 3, yielding a total score ranging from 0 to 21; higher scores indicate poorer sleep quality.

#### 2.2.4. Resilience

Resilience was assessed using the Connor–Davidson Resilience Scale (CD-RISC). This instrument assesses the psychological attributes essential for navigating stress, setbacks, and adversity, and is widely recognized as a robust measure of resilience [23,24]. The scale consists of 25 items rated on a 5-point Likert scale, with total scores ranging from 0 to 100; higher scores indicate greater resilience. Adapted for the Chinese cultural context [19], the scale integrates three dimensions: (1) Tenacity, which assesses the ability to remain determined, goal-oriented, and persistently engaged amidst stress and adversity; (2) Strength, which examines the capacity to mobilize internal and external resources, including emotional regulation and the efficacy of acquiring social support; and (3) Optimism, which reflects a positive cognitive tendency towards difficulties, maintaining a dialectical perspective and positive expectations for the future. By synthesizing scores from these three dimensions, the scale offers a robust and objective measure of participants’ psychological robustness and adaptive potential in response to physiological or environmental challenges.

### 2.3. Quality Control

To ensure data quality and accuracy, the survey protocol and field procedures were standardized. Prior to data collection, teacher–investigators received systematic training, including the use of standardized instructions, thorough familiarization with questionnaire content, and careful attention to key administration details. A strict data-cleaning procedure was implemented during preprocessing: responses were checked item-by-item for logical inconsistencies, missing data, erroneous information, and ambiguous answers; records were re-checked or removed as needed to maintain data validity. Throughout data collection, anonymity and confidentiality were emphasized, and participants were informed that data would be used for research purposes only, with efforts made to minimize potential biases during standardized administration.

### 2.4. Data Analysis

Statistical analyses were conducted using SPSS 30.0 (version 30.0; IBM Corp., Armonk, NY, USA) and Mplus 8.3 (version 8.3; Muthén & Muthén, Los Angeles, CA, USA). First, common method bias was assessed prior to model testing. Because PA, sleep quality, and resilience were all self-reported, a single-factor model was specified in Mplus, with the PA indicator, seven PSQI component indicators, and three CD-RISC dimension indicators loading onto one latent factor. Model fit was evaluated using CFI, TLI, RMSEA (with 90% CI), and SRMR; acceptable fit was defined as CFI/TLI ≥ 0.90, RMSEA ≤ 0.08, and SRMR ≤ 0.08.

Second, SPSS 30.0 was used for descriptive statistics of demographics and key variables. Categorical variables are presented as *n* (%). Group differences across the General Sample, Fitness-Remediation Sample, and Rehabilitation Sample were tested using chi-square tests. Spearman correlations were computed to examine associations among PA, PSQI components, and CD-RISC dimensions, with correlation coefficients and significance levels reported.

For structural analyses, a mediation structural equation model (SEM) was estimated in Mplus 8.3. At the measurement level, sleep quality and resilience were modeled using their respective component/dimension indicators. PA was modeled as a single observed composite indicator according to the IPAQ-SF scoring protocol, as the present study focused on the overall association of PA with sleep quality and resilience rather than intensity-specific PA patterns. At the structural level, we tested the direct associations of PA with sleep quality and resilience, as well as the indirect association between PA and resilience through sleep quality. Model fit criteria followed the thresholds above. Mediation was tested using bootstrap resampling (5000 draws) with bias-corrected 95% confidence intervals; an indirect effect was considered significant when the confidence interval did not include zero. Grade, sex, ethnicity, and BMI were included as covariates.

Given that participants came from different course types, multi-group SEM was further conducted. Measurement invariance was examined sequentially for configural, metric, and scalar invariance, using changes in fit indices as criteria (ΔCFI < 0.01 and ΔRMSEA < 0.01). After establishing invariance, equality constraints were imposed on structural paths and models were compared to test group differences in key path coefficients and indirect effects. All tests were two-sided, with *p* < 0.05 considered statistically significant.

## 3. Results

### 3.1. Common Method Bias Test

Since data for PA, sleep quality, and resilience were collected via self-report measures, common method bias (CMB) was assessed. Following previous studies, a confirmatory factor analysis (CFA) was performed in Mplus, where all indicators (one for PA, seven for PSQI, and three for CD-RISC) were loaded onto a single latent factor. The results indicated a poor model fit: χ^2^ = 2319.3, df = 44, *p* < 0.001; RMSEA = 0.159; CFI = 0.718; TLI = 0.648; SRMR = 0.132. These fit indices failed to meet the recommended psychometric thresholds, suggesting that a single common factor did not adequately account for the covariance among the study variables. However, because all core variables were self-reported and no marker variable was included in the original survey design, residual common method bias cannot be fully excluded.

### 3.2. Participant Characteristics

Table 1 presents the demographic characteristics and variable distributions across the groups. Chi-square tests revealed significant inter-group differences in sex distribution (χ^2^ = 48.10, *p* < 0.001), BMI categories (χ^2^ = 270.58, *p* < 0.001), and PA levels (χ^2^ = 90.85, *p* < 0.001). The within-group prevalence of obesity was highest in the Rehabilitation Sample (31.3%), followed by the Fitness-Remediation Sample (25.2%) and the General Sample (4.5%). Regarding PA levels, the within-group proportion of low PA was highest in the Rehabilitation Sample (34.9%), whereas the within-group proportion of vigorous PA was highest in the General Sample (43.3%).

**Figure 1:**
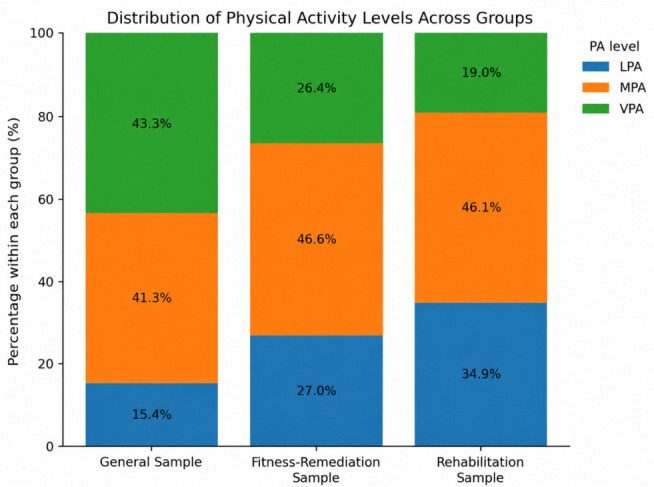
Distribution of physical activity levels across the three groups.

To improve the visual presentation of group differences in PA distribution, Figure 1 presents the proportions of low, moderate, and vigorous PA levels across the three groups. The Rehabilitation Sample showed the highest within-group proportion of low PA, whereas the General Sample showed the highest within-group proportion of vigorous PA.

### 3.3. Correlations Among Study Variables

Spearman correlation analysis (Table 2) showed that PA was negatively correlated with all sleep quality components, while sleep quality components were significantly and negatively associated with all resilience dimensions. Furthermore, PA was positively correlated with all resilience dimensions. All correlations reached statistical significance (*p* < 0.05).

### 3.4. Mediation Model Testing

A structural equation model (SEM) was constructed in Mplus 8.3 to examine the indirect association between PA and resilience through sleep quality, controlling for grade, sex, ethnicity, and BMI. The model demonstrated a good fit to the data: χ^2^ = 690.854, df = 254, and *p* < 0.001; CFI = 0.947; TLI = 0.940; RMSEA = 0.050; and SRMR = 0.047.

**Figure 2:**
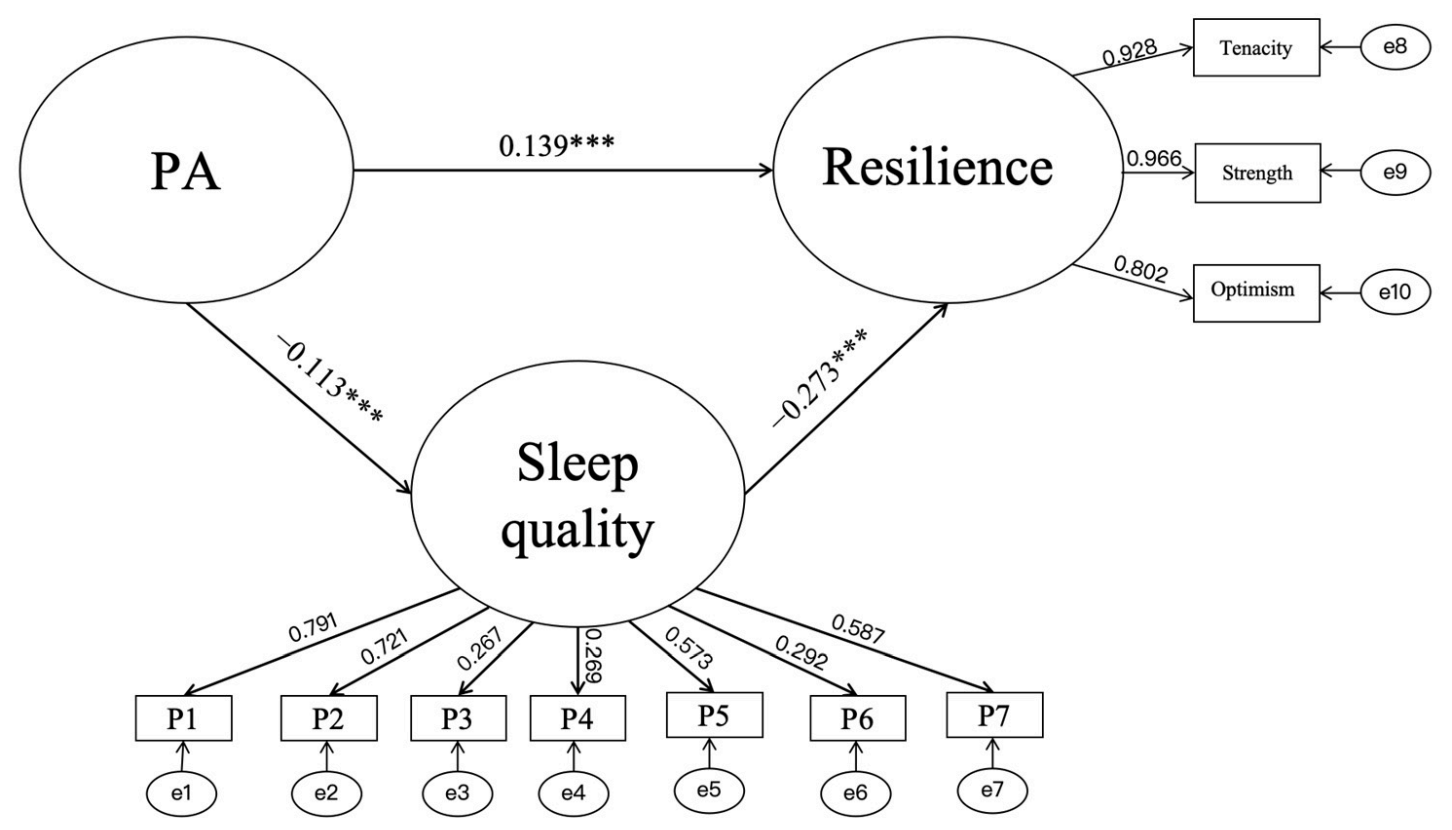
Indirect association model. *** *p* < 0.001.

Path analysis showed that PA was negatively associated with poorer sleep quality (β = −0.113, *p* < 0.001). Poorer sleep quality was negatively associated with resilience (β = −0.273, *p* < 0.001), whereas PA remained positively associated with resilience after controlling for sleep quality and covariates (β = 0.139, *p* < 0.001). Bootstrap results indicated a significant indirect association between PA and resilience through sleep quality (unstandardized estimate = 0.374, SE = 0.092, *p* < 0.001, 95% CI [0.199, 0.566]), accounting for approximately 18.2% of the total association (total effect = 2.055, *p* < 0.001). The total-sample mediation model is presented in Figure 2.

### 3.5. Multi-Group Analysis

To evaluate the model’s structural invariance across the three groups, we conducted multi-group measurement invariance testing. As shown in Table 3, the configural, metric, and scalar invariance models all exhibited acceptable fit (e.g., CFI = 0.934, RMSEA = 0.067). The differences in fit indices between nested models were minimal (ΔCFI = 0.004, ΔRMSEA = 0.003), satisfying the criteria for measurement invariance and allowing for cross-group structural path comparisons. Full standardized factor loadings and residual variances are presented in Supplementary Table S1.

**Figure 3:**
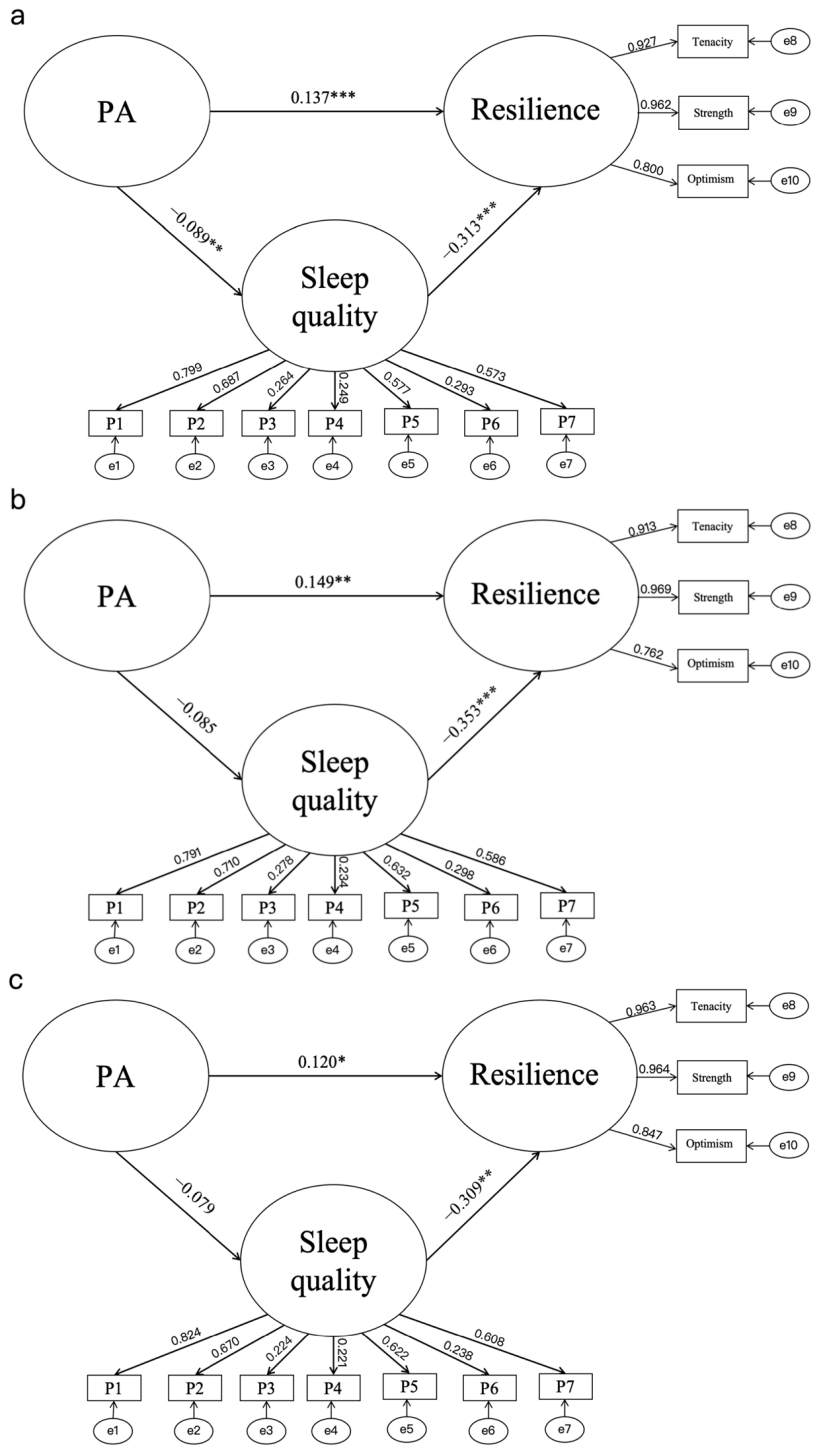
(**a**) Indirect association model of the General Sample; (**b**) indirect association model of the Fitness-Remediation Sample; (**c**) indirect association model of the Rehabilitation Sample. * *p* < 0.05, ** *p* < 0.01, *** *p* < 0.001.

Subsequent path comparisons revealed that the negative association between poorer sleep quality and resilience and the positive PA–resilience association were observed across all groups. However, the PA → poorer sleep quality path differed in statistical significance across groups. Specifically, this path was significant only in the General Sample (β = −0.089, Z = −2.633), but did not reach statistical significance in the Fitness-Remediation Sample (β = −0.085, |Z| = 1.377) or the Rehabilitation Sample (β = −0.079, |Z| = 1.746). Consequently, the indirect association through sleep quality was supported only in the General Sample. The group-specific indirect association models are presented in Figure 3.

## 4. Discussion

The present study aimed to examine the structural relationships among PA, sleep quality, and resilience, with a specific focus on testing the applicability and heterogeneity of this model across college student groups with different physical statuses. The empirical data generally supported the hypothesized “PA–Sleep Quality–Resilience” model. Path analysis of the total sample showed that PA was positively associated with resilience and showed an indirect association with resilience through sleep quality. This finding is broadly consistent with recent international evidence. A recent systematic review and meta-analysis reported a positive association between PA and psychological resilience among young students, while also noting limitations related to the number of included studies and the geographic concentration of existing evidence [25]. Compared with this broader evidence base, the present study further suggests that the PA–resilience association may remain observable across student groups with different physical statuses, whereas the indirect pathway through sleep quality may not be equally applicable to physically weak students.

Relevant theoretical frameworks provide possible explanations for these observed associations. First, the indirect association between PA and resilience through sleep quality is consistent with the Restorative Theory of Sleep [26]. From a physiological perspective, high-quality sleep is considered a critical window for the functional repair of the prefrontal cortex, a brain region serving as the neurobiological basis for emotional regulation and cognitive reappraisal [27]; previous evidence suggests that regular PA may be related to sleep through circadian and hormonal pathways, which may partly explain why PA and sleep quality were linked to resilience in the present model [28]. Recent studies among university students have also reported significant associations between PA and sleep quality, while further suggesting that this association may depend on psychological or behavioral moderators such as self-control [11,25,29,30]. Therefore, the significant indirect association observed in the total sample and General Sample is consistent with recent student-based evidence, whereas the non-significant PA–sleep quality paths in the Fitness-Remediation Sample and Rehabilitation Sample suggest that this association may be weaker or more context-dependent among physically weak students [31]. Second, the direct path coefficient from PA to resilience remained significant, a finding consistent with the Resilience Portfolio Model [32]. One possible interpretation is that physical exercise may serve as a controlled stressor and may provide opportunities to cope with physiological challenges in a relatively safe context. This interpretation is consistent with the observed positive PA–resilience association independent of the sleep pathway, although self-efficacy and mastery were not directly measured in this study.

However, through multi-group analysis, the descriptive results suggested that poorer physical status did not necessarily correspond to lower resilience; that is, objective physical weakness does not necessarily correspond to psychological fragility. Descriptive statistics showed that although students in the Rehabilitation Sample were at the lowest end of PA levels, their total scores for resilience, as well as scores for the tenacity and optimism dimensions, were significantly higher than those of students in the Fitness-Remediation Sample, who had relatively better physical conditions, and even higher than those of students in the General Sample. One cautious interpretation is that some students in the Rehabilitation Sample may have developed adaptive coping patterns while living with illness, injury, or functional limitations [33]. Since their participation in exercise is primarily to maintain bodily function and improve quality of life rather than to pursue performance or competition, this process often requires recognizing and accepting their own limitations while learning to adjust, set achievable goals, and persist within those limits [34]. From the perspective of Meaning-Making Theory, long-term adaptation to health-related constraints may involve accepting limitations, adjusting goals, and maintaining positive expectations under restricted conditions [35]. However, because meaning-making, illness acceptance, and goal adjustment were not directly measured in this study, this explanation should be regarded as a theoretical possibility rather than an empirically tested mechanism.

Further path comparison suggested that the indirect association through sleep quality differed across physical-status groups, although this pattern should be interpreted cautiously given the unequal group sizes and similar coefficient magnitudes for some paths. In the Fitness-Remediation Sample, no significant association was observed between PA and poorer sleep quality, suggesting that the indirect association through sleep quality was not supported in this group. This pattern may reflect the more complex exercise context experienced by students who had failed the university physical fitness test or were classified as physically underperforming. For these students, PA participation may occur partly in evaluative PE settings, where peer comparison, performance pressure, or concern about negative evaluation could potentially shape the psychological experience of exercise [36]. Social Evaluation Threat Theory provides a possible interpretive lens for understanding why PA may not be consistently associated with better sleep in this group. In such contexts, exercise may no longer be experienced purely as a restorative resource but is more likely associated with psychological stress experiences such as anxiety and rumination [37]. Nevertheless, because perceived evaluation pressure, exercise-related anxiety, and rumination were not directly assessed, this interpretation remains hypothetical and should be tested in future studies.

For the Rehabilitation Sample, the non-significant indirect association may be related to unmeasured physiological or clinical constraints. Although this group exhibited high levels of resilience, the data did not show a significant negative association between PA and poorer sleep quality. One possible explanation is that unmeasured clinical factors, such as chronic pain, medication use, or disease-related sleep disturbances, may have influenced sleep quality in this group. Because these factors were not assessed, this interpretation should be considered exploratory [38]. However, it is worth noting that the direct path of “PA–Resilience” remained significant across all three groups. This cross-group consistency suggests that even when the indirect association through sleep quality was not statistically supported, PA may still be associated with resilience through other psychological or social processes, such as bodily control, social connection, or active engagement [39]. These potential pathways require direct testing in future studies, particularly among students in the Rehabilitation Sample [8].

In summary, given the group-specific patterns in the associations among PA, sleep quality, and resilience, the present findings may inform the development of stratified student-support bundles from an operational perspective. A care bundle generally refers to a small set of evidence-based, feasible, independent, and monitorable practices designed for a specific population and care context [40]. Following this logic, the observed structural associations may be translated into differentiated support strategies for students with different physical statuses. For the General Sample, a basic support bundle may include regular PA promotion, sleep-hygiene education, and routine resilience screening. For the Fitness-Remediation Sample, the bundle may further emphasize individualized and progress-based exercise goals, reduction in social-evaluation pressure in PE settings, sleep-support counseling, and periodic monitoring of physical and psychological adaptation. For the Rehabilitation Sample, the bundle may prioritize adaptive PA prescriptions, sleep-hygiene management, medical or rehabilitation support when needed, and resilience monitoring. Such a bundle-informed framework may help translate theoretical associations into actionable strategies for university health promotion while allowing implementation fidelity and student adherence to be monitored in future intervention studies.

## 5. Limitations and Future Directions

The present study examined the associations among PA, sleep quality, and resilience across different college student groups and explored the indirect association through sleep quality. Several limitations should be acknowledged. First, although several demographic and anthropometric covariates were included in the SEM, the present study mainly focused on the structural associations among PA, sleep quality, and resilience. The effects of these covariates were not examined as primary research questions, and potential between-group differences in covariate effects were not further explored. Therefore, the interpretation of group-specific structural paths should consider the possible influence of demographic and anthropometric characteristics.

Second, in the present SEM analyses, grade, sex, ethnicity, and BMI were included as covariates to partially control for demographic and anthropometric confounding. However, several psychological, behavioral, and clinical confounders were not fully assessed. Variables such as baseline mental health status, academic stress, screen exposure, medication use, disease severity, chronic pain, exercise-related psychological experiences, meaning-making processes, illness acceptance, goal adjustment, and perceived social-evaluation threat may be associated with PA, sleep quality, and resilience [41]. Their omission may have influenced the observed associations, particularly in the Fitness-Remediation Sample and Rehabilitation Sample. Therefore, the theoretical explanations proposed in the Discussion should be interpreted as hypothesis-generating perspectives rather than confirmed mechanisms. Future studies should incorporate a broader set of psychological, behavioral, and clinical covariates and examine whether these factors modify or explain the group-specific associations among PA, sleep quality, and resilience. Longitudinal or intervention designs with more comprehensive covariate assessment would help clarify the stability and practical implications of these associations.

## 6. Conclusions

This study identified group differences in resilience and sleep-quality characteristics among college students with different physical statuses and showed that the structural associations among physical activity, sleep quality, and resilience varied across groups. Physical activity was positively associated with resilience across all groups, whereas the indirect association through sleep quality was supported only in the General Sample. This pathway was not supported in the physically weak student groups, suggesting that higher physical activity may not necessarily be accompanied by better sleep quality in these populations. Therefore, resilience-related health promotion among college students should not rely on a one-size-fits-all exercise-based approach. Instead, stratified support strategies based on physical characteristics should be considered. For physically weak students, an integrated support system combining adaptive physical activity prescriptions and sleep-hygiene management may be more appropriate for supporting resilience and psychosomatic health.

## Figures and Tables

**Table 1:** Mean differences in main study variables between groups.

		General Sample		Fitness-Remediation Sample		Rehabilitation Sample		*X^2^*	*p*
	Total	*n*	%	*n*	%	*n*	%		
Gender									
Male	1144	775	67.74%	235	20.54%	134	11.71%	48.10	<0.001
Female	896	729	81.36%	106	11.83%	61	6.81%		
Grade									
First	1015	762	75.07%	161	15.86%	92	9.06%	1.87	0.387
Second	1025	742	72.39%	180	17.56%	103	10.05%		
Nation									
Han	1854	1369	73.84%	308	16.61%	177	9.55%	0.17	0.919
Others	186	135	72.58%	33	17.74%	18	9.68%		
BMI									
Low	370	314	84.86%	35	9.46%	21	5.68%	270.58	<0.001
Normal	1132	914	80.74%	145	12.81%	73	6.45%		
Overweight	324	209	64.51%	75	23.15%	40	12.35%		
Obesity	214	67	31.31%	86	40.19%	61	28.50%		
PA									
LPA	392	232	59.18%	92	23.47%	68	17.35%	90.85	<0.001
MPA	870	621	71.38%	159	18.28%	90	10.34%		
VPA	778	651	83.68%	90	11.57%	37	4.76%		

**Table 2:** Correlations between study variables.

	PA	*p1*	*p2*	*p3*	*p4*	*p5*	*p6*	*p7*	*r1*	*r2*	*r3*
PA	1										
p1	−0.07 **	1									
p2	−0.06 **	0.56 **	1								
p3	−0.02 **	0.22 **	0.13 **	1							
p4	−0.05 **	0.17 **	0.20 **	0.27 **	1						
p5	−0.04 **	0.45 **	0.43 **	0.12 **	0.12 **	1					
p6	−0.02 **	0.20 **	0.19 **	0.04 **	0.07 **	0.16 **	1				
p7	−0.08 **	0.46 **	0.33 **	0.17 **	0.08 **	0.39 **	0.14 **	1			
r1	0.14 **	−0.20 **	−0.15 **	−0.04 **	−0.03 **	−0.18 **	−0.02 **	−0.26 **	1		
r2	0.14 **	−0.22 **	−0.17 **	−0.03 **	−0.02 **	−0.20 **	−0.06 **	−0.27 **	0.88 **	1	
r3	0.11 **	−0.20 **	−0.15 **	−0.01 **	−0.01 **	−0.17 **	−0.03 **	−0.21 **	0.74 **	0.75 **	1

p1–p7: indicators of sleep quality; r1–r3: indicators of resilience; ** *p* < 0.01.

**Table 3:** Multi-group invariance testing across samples.

Model	χ^2^	df	CFI	TLI	RMSEA	SRMR	Δχ^2^	Δdf	ΔCFI	ΔRMSEA
General	283.578	74	0.964	0.954	0.043	0.035	--	--	--	--
Fitness-Remediation	146.217	74	0.948	0.933	0.053	0.05	--	--	--	--
Rehabilitation	196.891	74	0.934	0.922	0.067	0.078	--	--	--	--
Configural Invariance	400.622	102	0.962	0.950	0.066	0.049	--	--	--	--
Metric Invariance	417.439	114	0.962	0.955	0.063	0.051	16.817	12	0	0.003
Scalar Invariance	465.151	134	0.958	0.958	0.060	0.053	47.712	20	0.004	0.003

## Data Availability

The data presented in this study are available on request from the corresponding author. The data are not publicly available due to privacy and ethical restrictions.

## Supplementary Materials

The following supporting information can be downloaded at https://www.mdpi.com/article/10.3390/healthcare14152255/s1, Table S1: Standardized factor loadings and residual variances of the scalar invariance model.
