# Supplementary material for: Structural Relationships Among Physical Activity, Sleep, and Resilience: A Multi-Group Structural Equation Modeling (SEM) Study Among Physically Weak College Students
Source: Healthcare (Basel). 2026 Jul 23;14(15):2255. doi: 10.3390/healthcare14152255 (PMC13465508; doi:10.3390/healthcare14152255)
Supplement: Supplementary file 1 [file healthcare-14-02255-s001.zip › healthcare-4219236-supplementary.pdf]

Supplementary Table S1. Standardized factor loadings and residual variances of the scalar invariance model

Latent variable	Indicator	General loading	Fitness-Remediation loading	Rehabilitation loading	General residual variance	Fitness-Remediation residual variance	Rehabilitation residual variance
Resilience	R1	0.926	0.912	0.962	0.143	0.168	0.074
Resilience	R2	0.962	0.975	0.965	0.075	0.050	0.069
Resilience	R3	0.799	0.765	0.847	0.362	0.415	0.282
Sleep quality	P1	0.799	0.790	0.826	0.362	0.376	0.318
Sleep quality	P2	0.687	0.712	0.673	0.528	0.494	0.548
Sleep quality	P3	0.264	0.276	0.225	0.931	0.924	0.949
Sleep quality	P4	0.248	0.232	0.220	0.939	0.946	0.952
Sleep quality	P5	0.578	0.630	0.623	0.666	0.603	0.612
Sleep quality	P6	0.292	0.296	0.238	0.915	0.912	0.944
Sleep quality	P7	0.575	0.584	0.613	0.669	0.659	0.624

Note. R1–R3 indicate the three dimensions of resilience; P1–P7 indicate the seven PSQI components.

Values are standardized estimates from the scalar invariance model.
